# Context matters

**DOI:** 10.1007/s10683-017-9546-z

**Published:** 2017-10-25

**Authors:** Wenting Zhou, John Hey

**Affiliations:** Department of Economics, University of York, Heslington, York, YO10 4GA UK

**Keywords:** Decision making, Experimental design, Experimental methods, Preference measures, Risk taking, D81, C91

## Abstract

Eliciting the level of risk aversion of experimental subjects is of crucial concern to experimenters. In the literature there are a variety of methods used for such elicitation; the concern of the experiment reported in this paper is to compare them. The methods we investigate are the following: Holt–Laury price lists; pairwise choices, the Becker–DeGroot–Marschak method; allocation questions. Clearly their relative efficiency in measuring risk aversion depends upon the numbers of questions asked; but the method itself may well influence the estimated risk-aversion. While it is impossible to determine a ‘best’ method (as the truth is unknown) we can look at the differences between the different methods. We carried out an experiment in four parts, corresponding to the four different methods, with 96 subjects. In analysing the data our methodology involves fitting preference functionals; we use four, Expected Utility and Rank-Dependent Expected Utility, each combined with either a CRRA or a CARA utility function. Our results show that the inferred level of risk aversion is more sensitive to the elicitation method than to the assumed-true preference functional. Experimenters should worry most about context.

## Introduction

Risk attitude is a crucial factor influencing economic behaviour. As a consequence, experimenters are interested in eliciting the risk-attitude of their subjects. This can be done in two ways: either *directly*, using the context of a particular experiment to estimate the risk-aversion that best explains behaviour; or *indirectly*, eliciting risk aversion in a separate part of the experiment, and using the elicited value to explain behaviour in the main experiment. It is to those experimenters that use the latter approach to whom this paper is addressed.

Economic theory posits that decisions under risk depend on how people evaluate, and hence decide between, risky lotteries. By these we mean lotteries where the outcomes are risky, and where the probabilities are known. Clearly how people evaluate lotteries depends not only on the lotteries, but also on the preference functionals of the decision-maker (DM). In the literature there are a number of proposed preference functionals, the best-known of which is the Expected Utility functional. All of these embody the idea of an underlying utility function *u*(.); it is the degree of concavity of this when it is defined over money that indicates the degree of risk-aversion. It is this that we are trying to elicit.

There are a number of methods that are used in the literature to elicit risk aversion. Possibly the most popular is that known as the *Holt*–*Laury Price List*, introduced by Holt and Laury (2002), and which we will refer to as HL. While the detail may vary from application to application, the basic idea is simple: subjects are presented with an ordered list of pairwise choices and have to choose one of each pair. The list is ordered in that one of the two choices is steadily getting better or steadily getting worse as one goes through the list. There are many variants on the basic theme: sometimes one of the two choices is a certainty, and that is getting better or worse through the list; sometimes either just one or both of the choices are risky choices and one of them is getting better or worse through the list. Because of the ordered nature of the list, subjects *should* choose the option on one side up to a certain point thereafter choosing the option on the other side. Some experimenters force subjects to have a unique switch point; others leave it up to subjects.^1^

A second method is to give a set of *Pairwise Choices*, but separately (not in a list) and not ordered. We will refer to this as PC. Typically the pairwise choices are presented in a random order. This has been used by Hey and Orme (1994), amongst many others. Some argue that this method, whilst being similar to that of Price Lists, avoids some potential biases associated with ordered lists. Usually the pairwise choices are chosen such that they are distributed over one or more Marschak-Machina triangles.^2^

A method which is elegant from a theoretical point of the view is the Becker–DeGroot–Marschak mechanism proposed by Becker et al. (1964). This we will later denote by Lottery Choice (LC) because of the way that we implement it. The method centres on eliciting the value to a subject of a lottery—if we know the value that a subject places on a lottery with monetary outcomes, we can deduce the individual’s attitude to risk over money. There are two variants of this mechanism that are used in the literature: one where the DM is told that they own the lottery, and hence have the right to play it out or to sell it; and one where the DM is offered the chance to buy the lottery, and, if so, to then play out the lottery. The subject’s valuation of the lottery as a potential seller is the *minimum* price for which they would be willing to sell it, while the subject’s valuation of the lottery as potential buyer is the *maximum* price for which they would be willing to buy it. Here we describe the mechanism as it relates to a potential buyer—the mechanism is the same, *mutatis mutandis*, if it relates to a potential seller. The subject is asked to state a number; then a random device is activated, which produces a random number between the lowest amount in the lottery and the highest amount. If the random number is less than the stated number, then the subject buys the lottery at a price equal to the random number (and then plays out the lottery); if the random number is greater, then nothing happens and the subject stays as he or she was. If^3^ the subject’s preference functional is the expected utility functional then it can be shown that this mechanism is incentive compatible and reveals the subject’s true evaluation of the lottery. Analytically it is equivalent to a Second Price Auction. The problem is that subjects do seem to have difficulty in understanding this mechanism, and a frequent criticism is that subjects understate their evaluation when acting as potential buyers and overstate it when acting as potential sellers. So in our experiment we implement this mechanism in a new way, which, we hope, is easier for subjects to understand, and which we will explain later.

The Allocation method, which we shall denote by AL, was originally pioneered by Loomes (1991). It was then revived by Andreoni and Miller (2002) in a social choice context, and later by Choi et al. (2007) in a risky choice context. This method involves giving the subject some experimental money to allocate between various states of the world, with specified probabilities for the various states, and, in some implementations, with given exchange rates between experimental money and real money for each of the states. This method seems easier for subjects to understand than BDM.

This paper is a follow-up, and complement to, the previous literature and, in particular, the paper by Loomes and Pogrebna (2014), in which the authors compare three of the elicitation methods described above—specifically Holt–Laury price lists, Ranking and Allocations. Our paper complements theirs, not only in the elicitation methods we consider, but also in that our experimental design (and crucially the numbers of questions asked for each method), as well as the data analysis, are completely different. We also consider a slightly different set of elicitation methods.

The purpose of this paper is to report on the results of an experiment in which subjects were asked to perform each of the four methods described in Sect. 1 above. The paper is organised as follows. We start with a literature review of the various elicitation methods in Sect. 2, and a survey of previous experimental results in Sect. 3. In Sect. 4 we describe how our experiment was organised and how the various methods were implemented in it, giving more detail about each of the methods. As we adopt an econometric methodology of fitting preference functionals to the data, we specify, in Sect. 5, the preference functionals that we fit to the data and describe the functional forms that we assume, and the parameters in them that we estimate. In Sect. 6, we describe how we analysed the data, detailing the stochastic assumptions that we make. Section 7 contains the results. In Sect. 8 we discuss what else we might have done; and Sect. 9 concludes.

## Review of the various elicitation methods

One clear difference between the methods is in the information that the answers give. Pairwise Choices (on which Price Lists are built) merely tell us which of two lotteries is preferred, but not by how much. In contrast both the LC and AL give us a continuous measure, which is (should be) the outcome of an optimising decision. This suggests that the latter two might be more informative, though of course more cognitive effort might have to be expended by the subjects. A discussion of the various methods can be found in Charness et al. (2013). A complete list, with links to descriptions, is given in Table 1. We briefly comment on them below.

**Table 1:** Elicitation methods

Allocation	Loomes (1991)
Angling risk task	Pleskac (2008)
Balloon analog risk task	Lejuez et al. (2002)
Becker–DeGroot–Marschak mechanism	Becker et al. (1964)
Bomb risk elicitation task	Crosetto and Filippin (2013)
Columbia card task	Figner et al. (2009)
Cups task	Levin and Hart (2003)
Deal or no deal	Deck et al. (2008)
Devil’s task	Slovic (1966)
Distribution builder	Goldstein et al. (2008)
Dynamic experiments for estimating preferences: risk	Toubia et al. (2013)
Eckel and Grossman method	Eckel and Grossman (2002)
First price auction	Isaac and James (2000)
Gneezy and Potters method	Gneezy and Potters (1997)
Lowa gambling task	Bechara et al. (1994)
Multi-outcome risky decision task	Lopes and Oden (1999)
Pairwise choices	Hey and Orme (1994)
Price list	Holt and Laury (2002)
Ranking task	Carbone and Hey (1994)
Reyna and Ellis risk task	Reyna and Ellis (1994)
Risk-taking propensity measures	MacCrimmon and Wehrung (1985)
Sequential investment task	Frey et al. (2015)
Two-outcome risky decision task	Lauriola et al. (2007)

This table was built from this site provided by the Society for Judgment and Decision Making and augmented with other methods not listed there

We classify the various methods into four main categories (and variants on them), namely: pairwise choice, multi-item choice, ‘continuous’^4^ choice and ranking. We do not give details but instead categorise the types of the methods and the underlying idea behind them.

In the first category we can obviously include the PC method and the Holt–Laury price list method (which comprises sets of ordered pairwise choices). The ‘Cups Task’, the ‘Reyna and Ellis Risk Task’ and the ‘Risk-taking Propensity Measures’ are special cases of pairwise choice in which one of the lotteries is a certainty. The ‘Two-Outcome Risky Decision Task’ is also a special case where both lotteries are two-outcome lotteries but one is risky while the other is ambiguous.^5^ The ‘Deal or No Deal Method’ and the ‘Dynamic Experiments for Estimating Preferences’ are dynamic versions of pairwise choice.

In the multi-option category there is the Eckel and Grossman method (with choice over 5 lotteries) and the ‘Iowa Gambling Task’ (with dynamic choice over four options with initially unknown probabilities).

In the ‘continuous’ choice category, we start with five methods that have strong similarities: the ‘Angling Risk Task’, the ‘Balloon Analog Risk Task’, the ‘Columbia Card Task’, the ‘Devil’s Task’ and the ‘Sequential Investment Task’. They all share the same basic idea: subjects have to choose a probability; then either some random event does occur with that probability or it does not occur with the residual probability; if it does occur the subject gets nothing, and, if it does not occur, the subject gets paid some increasing function of the probability. The chosen probability is an indicator of risk aversion. In an obvious sense the subject is choosing a distribution of his or her payment. The same is true with the Allocation method (which we have described above), the Becker–Degroot–Marschak method (which we have described above and which is analytically equivalent to a Second Price Auction), the ‘Distribution Builder’ task (in which subjects are asked to state a preferred distribution), the First Price Auction method (in which subjects have to state their valuation of a lottery), the Gneezy and Potters method (in which subjects are asked to allocate some money between a safe asset and a risky asset), and the ‘Sequential Investment Task’.

The ranking method offers subjects a choice of lotteries and asks them to rank them in order of preference (with an appropriate incentive mechanism). This has been used by Bateman et al. (2006) and Loomes and Pogrebna (2014), in addition to Carbone and Hey (1994).

## Previous experimental findings

Table 2 summarises previous experimental work comparing different elicitation methods. One recurring message from these previous studies is that there is little correlation in the measured degree of risk aversion across different methods. However there is one outstanding feature of most of this literature apparent from Table 2: namely the very small number of observations per subject: in most of them (16 out of the 23 excluding the present paper) only one task is implemented for each subject. So for each method there is only one observation. Moreover, in most of these (depending upon the elicitation method), this observation can lead only to an interval estimate of risk-aversion. In stark contrast, our paper has many tasks for each elicitation method and we can get a point estimate of risk attitude. Loomes and Pogrebna come closest to what we do, but they make a different use of the data, carrying out comparisons across tasks within an elicitation method. In contrast we use all our data from each method to estimate risk-aversion. True, it may be the case, that with large numbers of tasks, subjects get fatigued and hence possibly noisier, but offsetting that is the increased precision from having more observations.

**Table 2:** Previous experimental investigations of different elicitation methods

	Number of subjects	Allocation	Balloon analog risk task	Becker–DeGroot–Marschak	Bomb risk elicitation	Deal or no deal	Eckel and Grossman	First price auction	Gneezy and Potters	Holt Laury	Pairwise choice	Ranking
Charness and Viceisza (2012)	91								46(1)	45(1)		
Crosetto and Filippin (2016)	350				88(1)		88(1)		86(1)	88(1)		
Dave et al. (2010)	881						881(1)			881(1)		
Deck et al. (2008)	75					75(1)				75(1)		
Deck et al. (2013)	203		203(1)			203(1)	203(1)			203(1)		
Harbaugh et al. (2010)	128			128(6)							128(6)	
Isaac and James (2000)	34			34(4)				34(40)				
Loomes and Pogrebna (2014)	423	423(13)								423(5)		423(2)
Reynaud and Couture (2012)	30						30(1)			30(1)		
Zhou and Hey (this paper)	96	96(81)		96(54)						96(48)	96(80)	

The numbers in the table indicate the number of subjects with that elicitation method and (in brackets) the number of problems within that method

There are slight differences in the way that different methods were implemented in these papers. For example the number of rows in the price lists of HL (usually 10 or 11), but these are relatively unimportant. There is a common theme to the way that the data is analysed in many of these papers: usually it is assumed that the preference functional of the subjects is CRRA; under this assumption, intervals for the subjects’ risk aversions can be derived from the subjects’ decisions. This is then compared across elicitation methods. Some papers go further—trying to fit a CRRA preference functional to the data. One example is Crosetto and Filippin (2016). To do this they have to convert their one observation from each method into several observations. Take, for example HL. They have one price list with 10 rows and thus 10 ‘decisions’. They then interpret these in their econometrics as 10 *independent* observations. We (as will be seen) go to the opposite extreme—interpreting them as completely dependent—our view being that subjects will choose a ‘switch point’ and then fill in the table. It is not clear which is the correct assumption. Crosetto and Filippin (2016) go even further with their Gneezy and Potters data where they say that “we transformed the GP data into 40 binary choices”—assumed to be independent choices.

Some papers estimate an average risk-aversion index (rather than estimating subject-by-subject) and some try to explain this with various demographics and control variables.

Crosetto and Filippin (2016) also include something particularly interesting when they write: “We show by means of a simulation exercise that part of the often observed heterogeneity of estimates across tasks is due to task-specific measurement error induced by the mere mechanics of the tasks.” This, we suspect, is due to the way that the data from the different methods is analysed—using just one task to elicit an (interval) estimate of risk-aversion. Our method avoids this possible bias as we estimate across a number of tasks.

We should note in passing that the ‘framing’ of the different elicitation methods appears to have an influence on the elicited values. This is well-documented by Weber et al. (2002) and Lévy-Garboua et al. (2012). It might seem that this is only marginally relevant to this paper, though it could be argued that different methods are simply one method differently framed.

Although much research about the relationship between elicitation methods and elicited risk preferences has already been carried out, we feel that our paper makes a positive contribution to the literature. Our paper is different, not only in the elicitation methods considered and contrasted, but also in our experimental design and in the number of tasks posed to subjects. We illustrated lotteries as pictures, rather than in words, believing that a pictorial representation aids understanding, with a consequent reduction in noise. We have a different way of implementing the Becker–DeGroot–Marschak method. Our data analysis also differs from virtually all of the previous literature: we fit preference functionals to the data. We use maximum likelihood techniques to estimate the parameters of our preference functionals. We consider not only EU (Expected Utility) preferences but also RD (Rank-Dependent Expected Utility), and also two different utility functions—CRRA and CARA. Thus we estimate different preference functionals, EU-CRRA, EU-CARA, RD-CRRA, and RD-CARA; and not only compare the four elicitation methods, but also the four preference functionals. We can do this as we have a lot more data than most studies.

## The experimental implementation

We investigated a subset of four of the 20 methods listed in Table 1. We wanted them to be different; many of these 20 ‘overlap’. We chose Holt–Laury (because of its popularity), Pairwise Choice (as it is simple), Becker–Degroot–Marschak (again because of its popularity) and Allocation (because we think that it is more informative than pairwise choice and easier to understand than BDM). These four cover many of the 20 of our review. Our experiment was in four parts, corresponding to these four methods, with different ordering of the parts for different subjects.^6^ We describe the four parts here. All parts of the experiment concerned lotteries. The complete set of tasks can be found in https://www.york.ac.uk/economics/exec/research/zhouandhey1/.

**Fig. 1:**
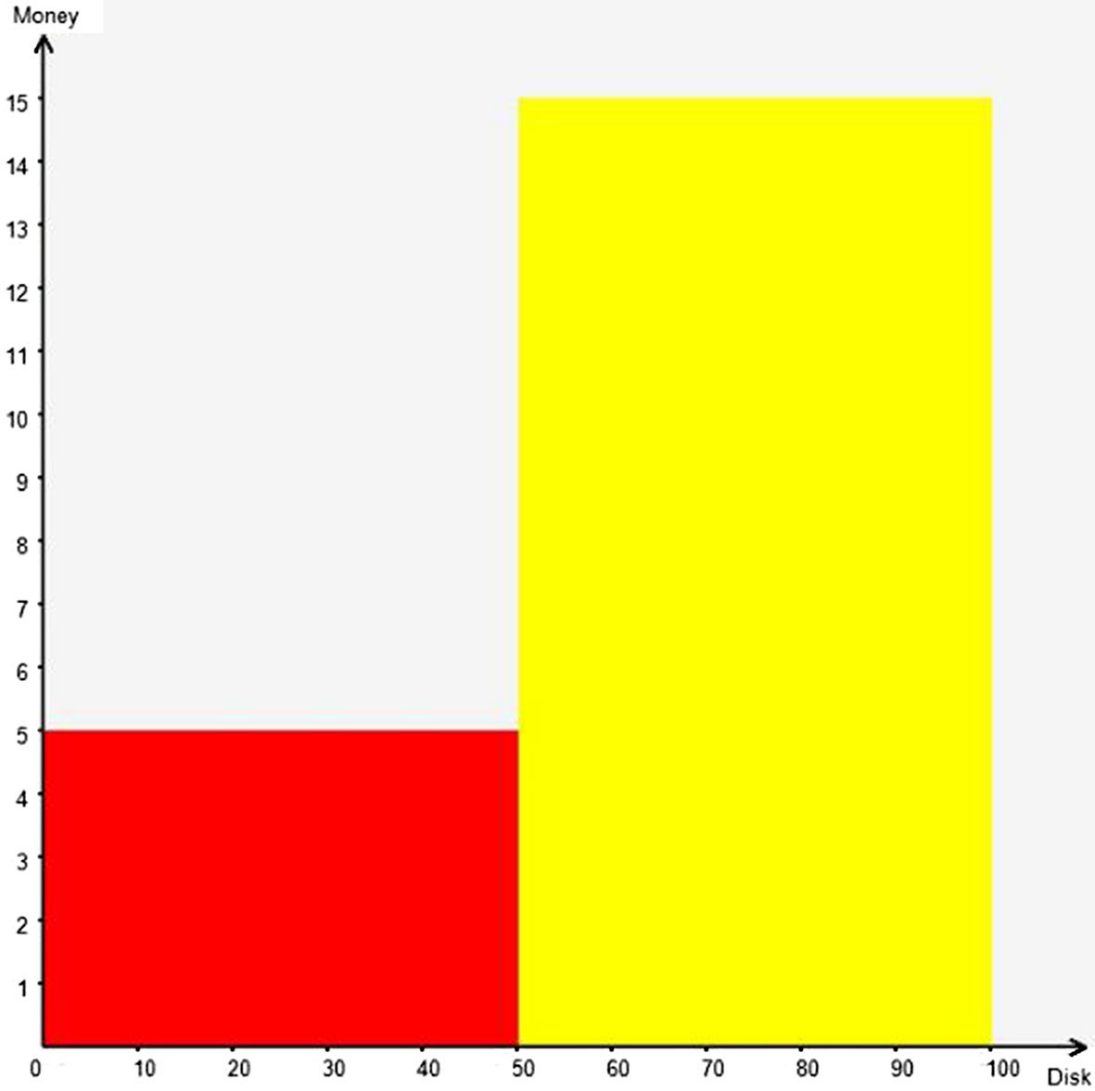
Representation of a lottery

Throughout the experiment, lotteries are visually displayed^7^ on the subjects’ computer screens in two dimensions, with the amount of money on the vertical axis and the chances on the horizontal axis. We used the random lottery incentive mechanism. If a particular lottery was chosen by this mechanism for paying out at the end of the experiment, the subject would draw a disk from a bag of 100 disks numbered from 1 to 100. The subject got paid the amount of money corresponding to the number on the disk. Let us give an example. Take the lottery in Fig. 1; this represents a lottery where there is a 1 in 2 (50 in 100) chance of gaining £5 and a 1 in 2 (50 in 100) chance of gaining £15. If this was played out at the end of the experiment, if the numbered disk was between 1 and 50 inclusive, the subject was paid £5; if it was between 51 and 100 inclusive the subject was paid £15. One of the possible advantages of this way of portraying lotteries is that the area indicates the expected value of the lottery.

**Fig. 2:**
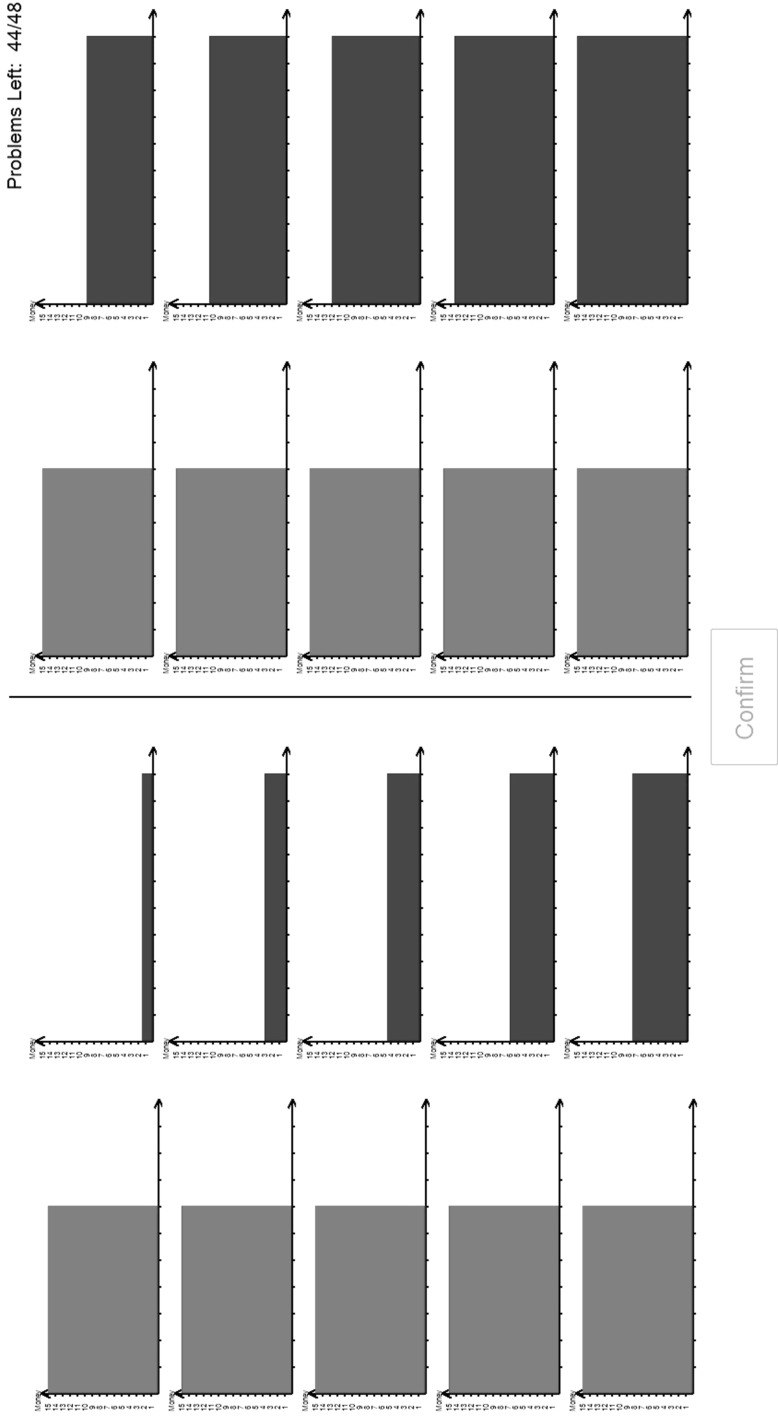

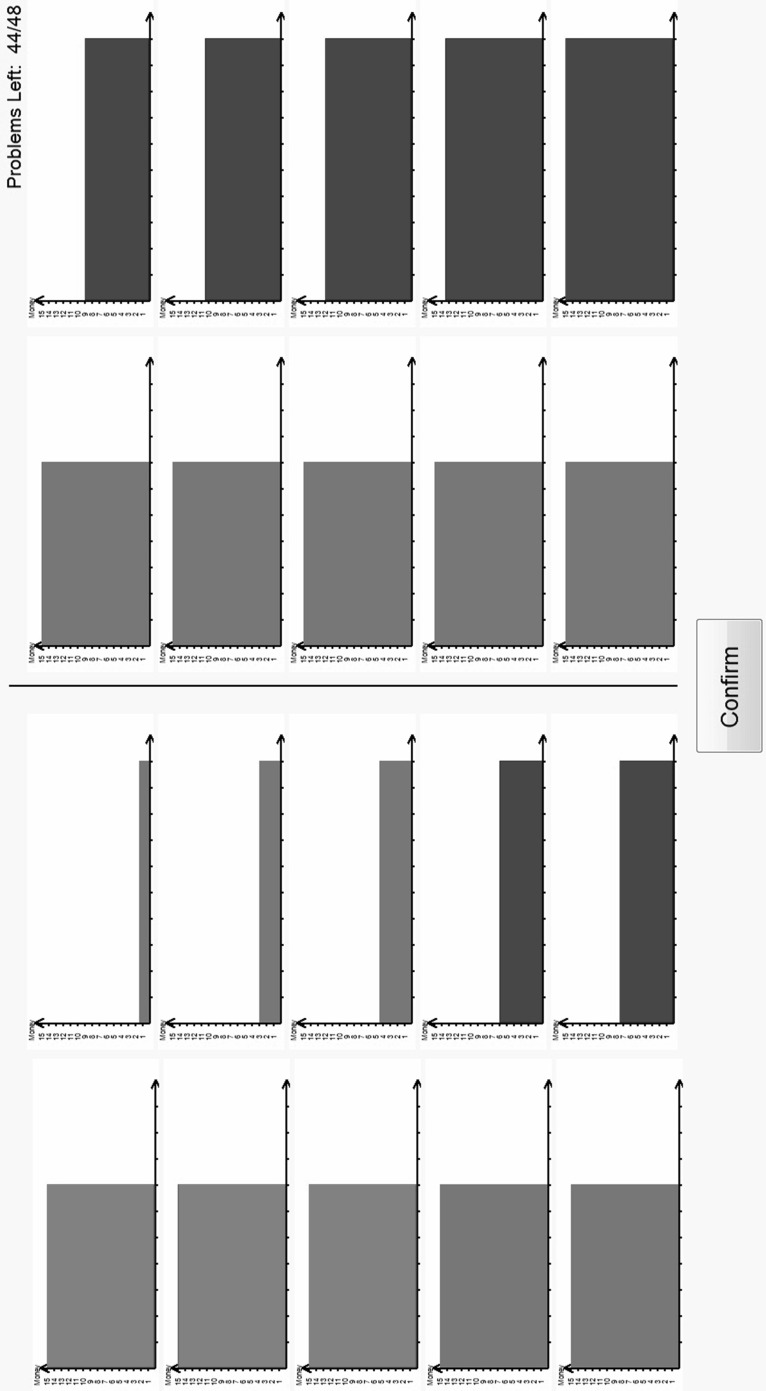
**a** A price list; **b** a completed price list

Part 1 presented a set of 48^8^ *Holt*–*Laury price lists* each containing 10 pairwise choices. An example is shown in Fig. 2a, b; Fig. 2a showing how it was first seen by the subject and Fig. 2b showing it after its possible completion by a subject. These are screen shots from the experimental software; they appeared full-screen in the experimental interface. In Fig. 2a, the thing that is staying constant is the lottery on the left (which is a 70% chance of £15 and a 30% chance of £0); the thing that is changing is that on the right—in this case a certainty—which increases from a certainty of £1.50 to a certainty of £15. Subjects were asked, for each pair in the list, to click on the preferred item; when doing so, the item on the other side turned grey. Figure 2b shows a possible set of responses—with the lottery being preferred to the certainty until the value of certainty reached £6. To avoid problems with subjects switching at several points^9^ within the list, the software forced subjects to choose a unique switching point. The 48 Price Lists spanned a variety of cases; details in https://www.york.ac.uk/economics/exec/research/zhouandhey1/. We denote this method HL (as it comes from Holt–Laury mechanism).

**Fig. 3:**
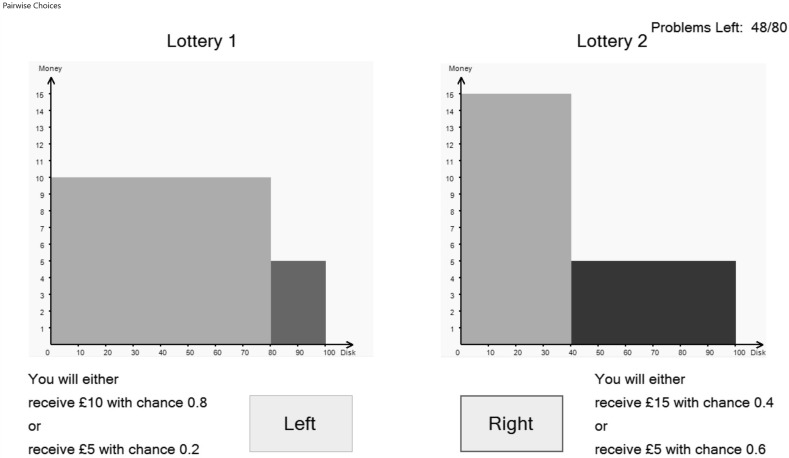
A pairwise choice

Part 2 asked subjects to respond to 80^10^ *pairwise choice* questions. An example is shown in Fig. 3.

In this pairwise choice, subjects had to choose between a lottery which give an 80% chance of £10 and a 20% chance of £5 and a lottery which give a 40% chance of £15 and a 60% chance of £5. The set of 80 pairwise choice questions spanned lotteries with outcomes of £0, £5, £10 and £15 with probabilities of 0, 0.2, 0.4, 0.6, 0.8 and 1.0; details in https://www.york.ac.uk/economics/exec/research/zhouandhey1/. We denote this method PC.

**Fig. 4:**
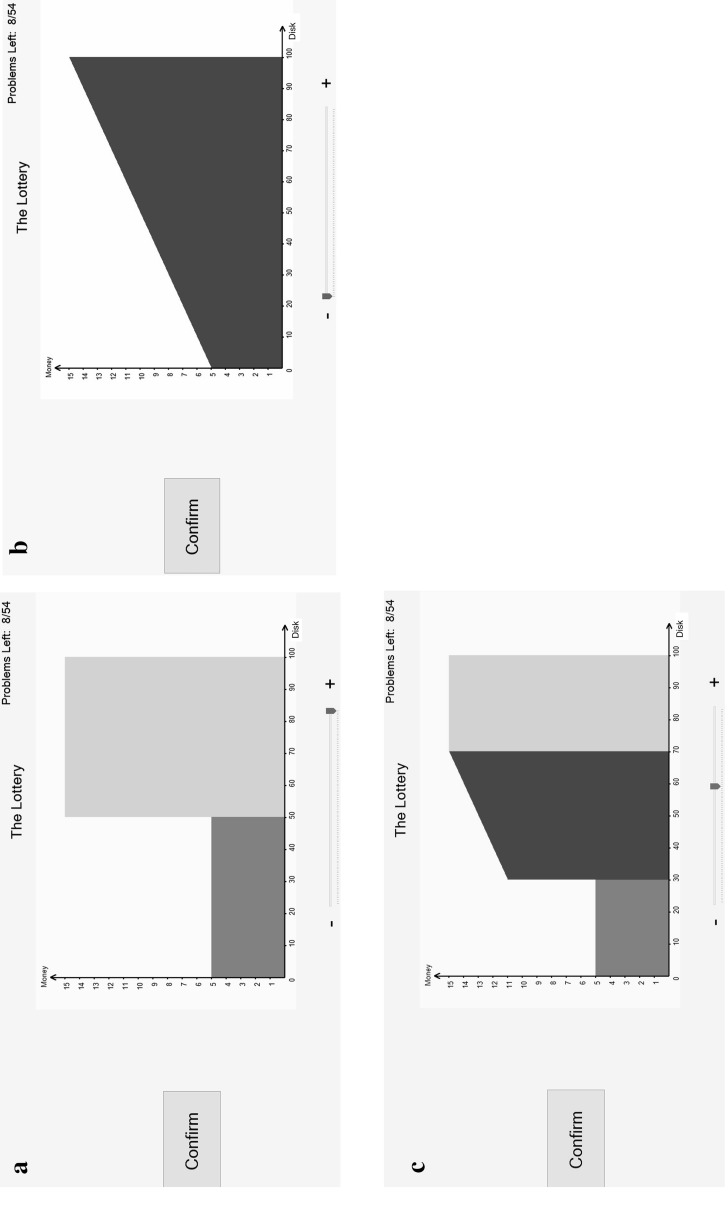
**a** The lottery (in the Becker-Degroot-Marschak mechanism); **b** the uniform distribution (over the range of the lottery); **c** a lottery choice

Part 3 asked subjects to respond to 54^11^ *Becker*–*DeGroot*–*Marschak* problems. Typically subjects are shown a lottery and asked to state their maximum willingness-to-pay or minimum willingness-to-accept for the lottery. Many experimenters have reported confusion among subjects with understanding this mechanism, so we adopted a new way of implementing it. Suppose that we want to find the subject’s certainty equivalent of a lottery which pays £*x* with probability *p* and £*y* with probability *1* − *p*, where *x* > *y.* The subject is asked to choose a number £*z.* We want *z* to be the certainty equivalent. To obtain this in an incentive compatible way,^12^ we could tell the subjects that a random number *Z* will be generated from a uniform distribution over the interval (*y*, *x*) and that they will get paid *Z* if *Z* > *z* and will get to play out the lottery if *Z* ≤ *z*. The optimal choice of *z* is the subject’s certainty equivalent of the lottery. Consider the implications in terms of what they are choosing: their choice of *z* implies the choice of a lottery, which is a *compound* of the original lottery and the uniform distribution. To illustrate this, consider the lottery in Fig. 4a, where the payoffs are £5 and £15. If they state *z* = *5* they get to play out the lottery; if they state *z* = *15*, they are opting for the lottery in Fig. 4b—that is a uniform^13^ distribution over (5,15); if they state some number in between, for example 11, they are opting for the lottery in Fig. 4c. As *z* is varied from 5 to 15, the lottery in Fig. 4c varies from that in Fig. 4a to that in Fig. 4b. We simply asked them to choose their preferred lottery; they did this by moving the slider below the graph and then clicking on ‘Confirm’. The implied value of *z* given by a choice of the lottery in Fig. 4c is 11; this is the observed certainty equivalent.

We feel that this is a simpler and more understandable implementation of the Becker–DeGroot–Marschak mechanism, and is a major contribution of our paper. We denote it by LC—Lottery Choice—as they are choosing their preferred lottery. The 54 LC problems spanned lotteries with outcomes of £0, £5, £10 and £15, and with probabilities ranging from 0.0 to 1.0 in steps of 0.1; details in https://www.york.ac.uk/economics/exec/research/zhouandhey1/.

**Fig. 5:**
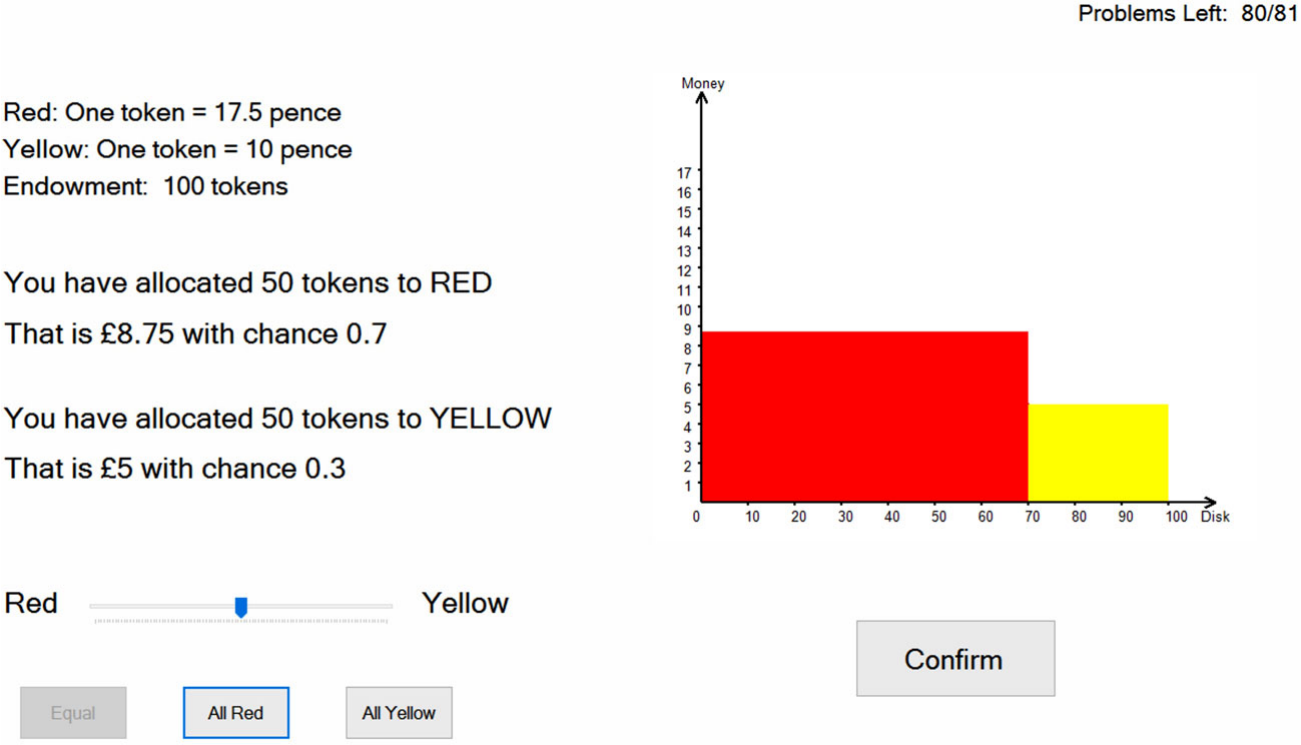
An allocation

Part 4 asked subjects to respond to 81 *allocation* problems.^14^ An example is shown in Fig. 5. In this example, the two states (red and yellow) have probabilities 0.7 and 0.3 respectively. Subjects have 100 tokens to allocate, and the exchange rates between tokens and money are 1 token = 17.5p for red, and 1 token = 10p for yellow. They made their allocation with the slider, with the figure showing the implied amounts of money (and their probabilities). The 81 allocation problems spanned probabilities ranging from 0 to 1 in steps of 0.1 with varying exchange rates; details in https://www.york.ac.uk/economics/exec/research/zhouandhey1/. We denote this method AL.

## Functional forms assumed

While we are primarily interested in the differences in the estimated risk attitudes between the different elicitation methods, in order to understand these differences we need to model behaviour and hence estimate the risk attitude. To model the behaviour we^15^ need to choose preference functionals. We do not know the preference functionals of our subjects, so we have to choose a set of such functionals and use our data to find the best-fitting one(s). We choose the most popular in the literature, namely Expected Utility (EU) and Rank Dependent expected utility (RD).

Let us denote by *V* the value to a subject of a 3-outcome lottery which pays *x*_*i*_ with probability *p*_*i*_ (for *i* = 1,2,…,3), and let us order the payoffs so that *x*_*1*_ ≥ *x*_*2*_ ≥ *x*_*3*_, then we have

$${\text{for}}\quad {\text{EU:}}\;u\left( V \right) = p_{1} u\left( {x_{1} } \right) + p_{2} u\left( {x_{2} } \right) + p_{3} u(x_{3} )$$

$${\text{for}}\quad {\text{RD:}}\; u\left( V \right) = w\left( {p_{1} } \right)u\left( {x_{1} } \right) + \left[ {w\left( {p_{1} + p_{2} } \right) - w\left( {p_{1} } \right)} \right]u\left( {x_{2} } \right) + \left[ {1 - w\left( {p_{1} + p_{2} } \right)} \right]p_{3} u(x_{3} )$$

In these expressions, *u*(.) is the underlying utility function of the subject and *w*(.) is the rank-dependent weighting function.

We need to specify the utility function *u*(.) which appears in both these functionals. We adopt both the constant Relative Risk aversion (RR) form and the constant Absolute Risk aversion (AR) form. These are given by

$${\text{for}}\quad {\text{RR:}}\;u\left( x \right) = x^{1 - r} /\left( {1 - r} \right), \quad r \ne 1;\quad \ln \left( x \right),\quad r = 1$$

$${\text{for}}\quad {\text{AR:}}\;u\left( x \right) = - \exp \left( { - rx} \right),\quad r \ne 0;\;x,\quad r = 0.$$

We note that in both cases *r* = *0* corresponds to risk-neutrality and increases in *r* imply greater risk aversion, but there is no mapping between the *r* for RR and that for AR. This is because the *r* in RR is a measure of *relative* risk-aversion, while the *r* in AR is a measure of *absolute* risk-aversion.

In fitting the RD specifications we also need to specify a *weighting function* for the probabilities. This we take to be of the following form (Tversky and Kahneman 1992):

$$w\left( p \right) = p^{g} /\left[ {p^{g} + \left( {1 - p} \right)^{g} } \right]^{1/g}$$

In the results that follow we fit, for all four elicitation methods, the four possible combinations of the two preference functionals and the two utility functions, using the obvious notation RREU, RRRD, AREU and ARRD. Essentially we want to see which of these best explains the data and we also want to see whether the estimated parameters differ across the elicitation methods; we do this on a subject-by-subject basis, as it is clear that subjects are different.

## Our stochastic assumptions and econometric methodology

We should comment on our econometric methodology, as it is different from that used by others. We treat subjects as different, so we analyse subject-by-subject.^16^ We also use simultaneously *all* the responses of the subjects on *all* problems of a particular elicitation method (and use them for estimation), rather than compare responses on particular problems. The latter is what Loomes and Pogrebna (2014) and many others have done. So, for example, in their Table 1 on their page 578, they look at the distribution of responses^17^ for particular decision tasks and compare these distributions across tasks. They note that the distributions are different across tasks, sometimes significantly so. This could be the case because of noise in subjects’ responses but they present no way of modelling this noise, though the use of a statistical test (in this case a Mann–Whitney test) does necessarily involve some implicit assumption about stochastics.

Another difference between our methodology and that of others is in the number of problems we present to our subjects: pre-experimental simulations show that one needs large numbers of observations to get precise estimates since there is a lot of noise in subjects’ behaviour. Unfortunately there is a downside to having a large number of problems if subject fatigue is increased and because the financial incentive per decision is reduced, we might get increased noise and decreased precision.

Our econometric methodology is to fit, for each of RREU, RRRD, AREU and ARRD, the models to the decisions of the subjects, for each of the four elicitation methods, and hence obtain estimates of the risk aversion index (and also the other parameters). We do this by Maximum Likelihood, using Matlab (the program is available in https://www.york.ac.uk/economics/exec/research/zhouandhey1/). To do this we need to make assumptions about the stochastic nature of the data. This arises from errors made by the subjects. We largely follow convention.

- HL: we assume that the subject calculates the utility difference between the two lotteries for each pair in the list, but makes an error in the calculation. Further, embodying the fact that the list is presented as a list, we assume that the subject *makes the same error* for each pair; we further assume that this error has a normal distribution with mean 0 and precision (the inverse of the standard deviation) *s.* Then the switch-point decision is taken on the basis of where this utility difference plus error changes from positive to negative or vice versa.
- PC: we assume that on each problem the subject calculates the utility difference between the two lotteries, but makes an error in this calculation; we further assume that this error is independent across problems and has a normal distribution with mean 0 and precision (the inverse of the standard deviation) *s.* So that the decision is taken on the basis of the sign of the utility difference plus error.
- LC: we assume that the subject calculates the certainty equivalent of the lottery, but makes an error in this calculation; we further assume that this error has a normal distribution with mean 0 and precision (the inverse of the standard deviation) *s.* So that the observed certainty equivalent is the subject’s true equivalent plus error.
- AL: we assume that the subject calculates the optimal allocation that he or should make, but makes an error in this calculation; we further assume that this error has a normal distribution with mean 0 and precision (the inverse of the standard deviation) *s.* So that the observed allocation is the optimal allocation plus error.

We note that the *s* in the HL and PC stories are on a different scale than the *s* in the PC and AL stories—the former being on utilities and the latter on money.

## Results

We had 96 subjects, who each completed all four parts of the experiment. For each subject and for each elicitation method, we attempted to fit the four models RREU, RRRD, AREU and ARRD to their decisions; so for each subject 16 models were estimated. This implies a total of 1536 estimations. In certain cases the estimation did not converge. This was for a variety of reasons which we discuss below. In the table below we enumerate these cases by elicitation method. It will be seen from this table that the ‘worst offender’ is PC. There were a total of 20 subjects where convergence was not obtained on at least one method. As the point of the paper is to compare different elicitation methods, we exclude all these 20 subjects from the analysis that follows [though an online appendix (https://www.york.ac.uk/economics/exec/research/zhouandhey1/) repeats parts of the analyses with all 96 subjects].

**Table:** 

Method(s)	Number of times not converged
Just LC	3
Just PC	9
Just HL	5
Both AL and LC	1
Both AL and PC	1
Both LC and PC	1

These cases of non-convergence took several forms: (1) where the subject was clearly either risk-neutral or risk-loving—in which cases the implied parameters are not unique; (2) where the estimation hit the bounds imposed by us on the parameters^18^; (3) where the subject appeared not to understand the tasks, or where the subject appeared to be responding randomly.

We present our results in two main parts. In Sect. 7.1 we present some summary statistics. Then in Sect. 7.2 we compare the estimated parameters across preference functionals for given elicitation methods; finally in Sect. 7.3 we compare the estimated parameters across elicitation methods for given preference functionals.

### Summary statistics

Table 3 presents some summary statistics.^19^ It is very clear from this that the estimated parameters vary widely across the different elicitation methods. For example, using AL the risk-aversion index elicited in the RR specifications is, on average, much higher that found with the other methods, and also has a much higher spread. It may well be that the elicitation method is affecting the way that subjects process the problems. For example the allocation method may be focussing subjects’ minds on what outcome they might obtain for different states of the world.

**Table 3:** Summary statistics

Stats	Method	RREU		RRRD			AREU		ARRD		
		*r*	*s*	*r*	*g*	*s*	*r*	*s*	*r*	*g*	*s*
Mean	PC	0.502	1.666	0.375	1.105	2.135	0.175	0.127	0.134	1.112	0.164
	AL	3.144	0.161	2.059	1.120	0.168	0.078	0.148	0.052	1.021	0.151
	LC	0.192	0.594	0.028	0.959	0.635	0.094	0.564	0.043	1.027	0.598
	HL	0.182	0.955	− 0.022	0.824	0.963	0.068	0.110	0.026	0.741	0.136
Median	PC	0.535	1.358	0.399	0.850	1.515	0.157	0.111	0.109	0.870	0.137
	AL	1.054	0.078	0.947	1.005	0.087	0.027	0.068	0.024	0.924	0.070
	LC	0.329	0.539	0.237	0.907	0.580	0.073	0.530	0.041	0.904	0.549
	HL	0.174	0.777	0.004	0.648	0.748	0.044	0.097	0.018	0.646	0.121
Standard deviation	PC	0.275	1.440	0.320	0.714	2.998	0.131	0.077	0.154	0.701	0.131
	AL	10.367	0.646	8.450	0.428	0.655	0.231	0.661	0.186	0.403	0.667
	LC	0.956	0.193	1.092	0.401	0.192	0.189	0.164	0.183	0.535	0.166
	HL	0.285	1.136	0.308	0.622	1.240	0.089	0.144	0.087	0.314	0.149

### A comparison of the estimated parameters across preference functionals^20^

The parameters of the various specifications are the risk-aversion index *r* (for both the EU and the RD functionals), the weighting function parameter *g* (for the RD functional) and the precision parameter *s* for all specifications. Some of these parameters are comparable across preference functionals (in that they have the same definition and interpretation) and some are not (like *r* with RR and *r* with RA—as the first measures the degree of *relative* risk aversion and the second the degree of *absolute* risk aversion). Table 4 shows the relationships between them for those that are comparable; Table 5 shows the relationships for those that are not comparable.

**Table 4:** A comparison of the estimated parameters across preference functionals (part 1)

Parameter	Method	*x*	*y*	*α*	*β*	*ρ*
*r*	PC	RREU	RRRD	− 0.121***	0.988	0.849
*r*	PC	AREU	ARRD	− 0.048***	1.035	0.875
*s*	PC	RREU	RRRD	0.538***	0.789***	0.795
*s*	PC	AREU	ARRD	0.046***	0.844***	0.879
*g*	PC	RRRD	ARRD	0.624***	0.442***	0.451
*r*	AL	RREU	RRRD	0.185**	0.639***	0.801
*r*	AL	AREU	ARRD	0.009***	0.528***	0.700
*s*	AL	RREU	RRRD	0.001	1.058***	0.987
*s*	AL	AREU	ARRD	− 0.001	1.037***	0.997
*g*	AL	RRRD	ARRD	0.373***	0.579***	0.617
*r*	LC	RREU	RRRD	− 0.179***	1.082	0.923
*r*	LC	AREU	ARRD	− 0.040***	0.884**	0.913
*s*	LC	RREU	RRRD	0.078***	0.938	0.941
*s*	LC	AREU	ARRD	0.055**	0.963	0.950
*g*	LC	RRRD	ARRD	0.095	0.975	0.731
*r*	HL	RREU	RRRD	− 0.174***	0.835**	0.773
*r*	HL	AREU	ARRD	− 0.030***	0.829***	0.848
*s*	HL	RREU	RRRD	− 0.111*	1.139**	0.890
*s*	HL	AREU	ARRD	0.008	1.210**	0.847
*g*	HL	RRRD	ARRD	0.421***	0.388***	0.769

This table is for where the parameters are comparable. The *α* (intercept) and *β* (slope) values are obtained from a regression of the estimated parameter value for the *y* preference functional against the estimated parameter value for the *x* preference functional. The *ρ* value is the correlation coefficient. If they produce the same estimates *α* should be zero and *β* should be unity

The hypotheses being tested are *α* = 0 and *β* = 1

Key: preference functionals: RREU: expected utility with cRRa utility function, AREU: expected utility with cARa utility function, RRRD: rank dependent with cRRa utility function, ARRD: rank dependent with cARa utility function

Elicitation methods: PC: pairwise choices, AL: alocations, LC: lottery choice (Becker–DeGroot–Marschak mechanism), HL: Holt Laury price list

* Significantly different (from 0 for *α* and from 1 for *β*) at 10%; ** at 5% and *** at 1%

**Table 5:** A comparison of the estimated parameters across preference functionals (part 2)

Parameter	Method	*x*	*y*	*α*	*β*	*ρ*
*r*	PC	RREU	AREU	− 0.036**	0.422***	0.889
*r*	PC	RREU	ARRD	− 0.073***	0.410***	0.731
*r*	PC	RRRD	AREU	0.056***	0.318***	0.779
*r*	PC	RRRD	ARRD	− 0.009	0.380***	0.788
*s*	PC	RREU	AREU	0.044***	0.051***	0.816
*s*	PC	RREU	ARRD	0.084***	0.043***	0.637
*s*	PC	RRRD	AREU	0.053***	0.040***	0.622
*s*	PC	RRRD	ARRD	0.074***	0.043***	0.678
*r*	AL	RREU	AREU	0.007***	0.020***	0.979
*r*	AL	RREU	ARRD	0.012***	0.012***	0.746
*r*	AL	RRRD	AREU	0.014***	0.019***	0.724
*r*	AL	RRRD	ARRD	0.008***	0.019***	0.968
*s*	AL	RREU	AREU	0.002	0.809***	0.901
*s*	AL	RREU	ARRD	0.002	0.834***	0.893
*s*	AL	RRRD	AREU	0.004	0.732***	0.873
*s*	AL	RRRD	ARRD	0.004	0.759***	0.872
*r*	LC	RREU	AREU	0.026**	0.260***	0.880
*r*	LC	RREU	ARRD	− 0.022*	0.247***	0.857
*r*	LC	RRRD	AREU	0.090***	0.137***	0.790
*r*	LC	RRRD	ARRD	0.039***	0.143***	0.851
*s*	LC	RREU	AREU	0.208***	0.600***	0.705
*s*	LC	RREU	ARRD	0.286***	0.525***	0.609
*s*	LC	RRRD	AREU	0.202***	0.571***	0.668
*s*	LC	RRRD	ARRD	0.229***	0.581***	0.671
*r*	HL	RREU	AREU	0.019***	0.267***	0.857
*r*	HL	RREU	ARRD	− 0.016**	0.231***	0.757
*r*	HL	RRRD	AREU	0.072***	0.204***	0.706
*r*	HL	RRRD	ARRD	0.031***	0.228***	0.806
*s*	HL	RREU	AREU	0.033***	0.070***	0.723
*s*	HL	RREU	ARRD	0.048***	0.085***	0.618
*s*	HL	RRRD	AREU	0.050***	0.048***	0.638
*s*	HL	RRRD	ARRD	0.060***	0.070***	0.648

The hypotheses being tested are *α* = 0 and *β* = 0

This is for where parameters are not comparable. The *α* (intercept) and *β* (slope) values are obtained from a regression of the estimated parameter value for the *y* preference functional against the estimated parameter value for the *x* preference functional. The *ρ* value is the correlation coefficient. The parameters should at least be positively related so that *β* should be positive

Key: see key for Table 4

* Significantly different (from 0) at 10%; ** at 5% and *** at 1%

Let us start with Table 4 where estimates *are* comparable. We can compare *g* across RRRD and ARRD, and similarly we can compare *s* across the various preference functionals. Also we can compare the *r* between RREU and RRRD, and between AREU and ARRD, though clearly if the true preference functional is Rank Dependent then assuming Expected Utility preferences may lead to bias. Table 4 reports the correlation (*ρ*) between the estimated parameters for different elicitation methods and the intercept (*α*) and slope (*β*) of a linear regression of one against the other. If they were consistently producing the same estimates then *α* should be zero, and *β* (and the correlation coefficient) should be one. Table 4 shows that the estimated values of *s*, across preference functionals, are generally not too far apart. For example, the estimated values of *s* (the precision parameter) using the AL method are very close whether we fit RREU or RRRD. This is less true for the estimated values of *g* (the weighting function parameter), though they are very similar using the LC method whether we fit RRRD or ARRD. However this is not always the case: for example, there are big differences between the estimated values of *g* using the PC method depending on whether we fit RRRD or ARRD. The estimated *r* values differ more markedly across the elicitation methods, though once again there are cases (using LC and comparing RREU and RRRD) where the fit is good. Even though it is difficult to summarise a whole table in one sentence, one could say that the correlations are all positive and reasonably large, and certainly larger than in Table 5 (comparisons across elicitation methods), which we shall come to shortly.

Some of the parameters are not comparable. Crucially the *r* parameter differs between the Constant Absolute Risk Aversion specification and the Constant Relative Risk Aversion specification in both what it measures and its scale; moreover there is no precise mapping between them. However increases in either imply a higher risk-aversion so that they should be positively related. Table 5 shows the results. Again the correlations are reasonably high.

**Fig. 6:**
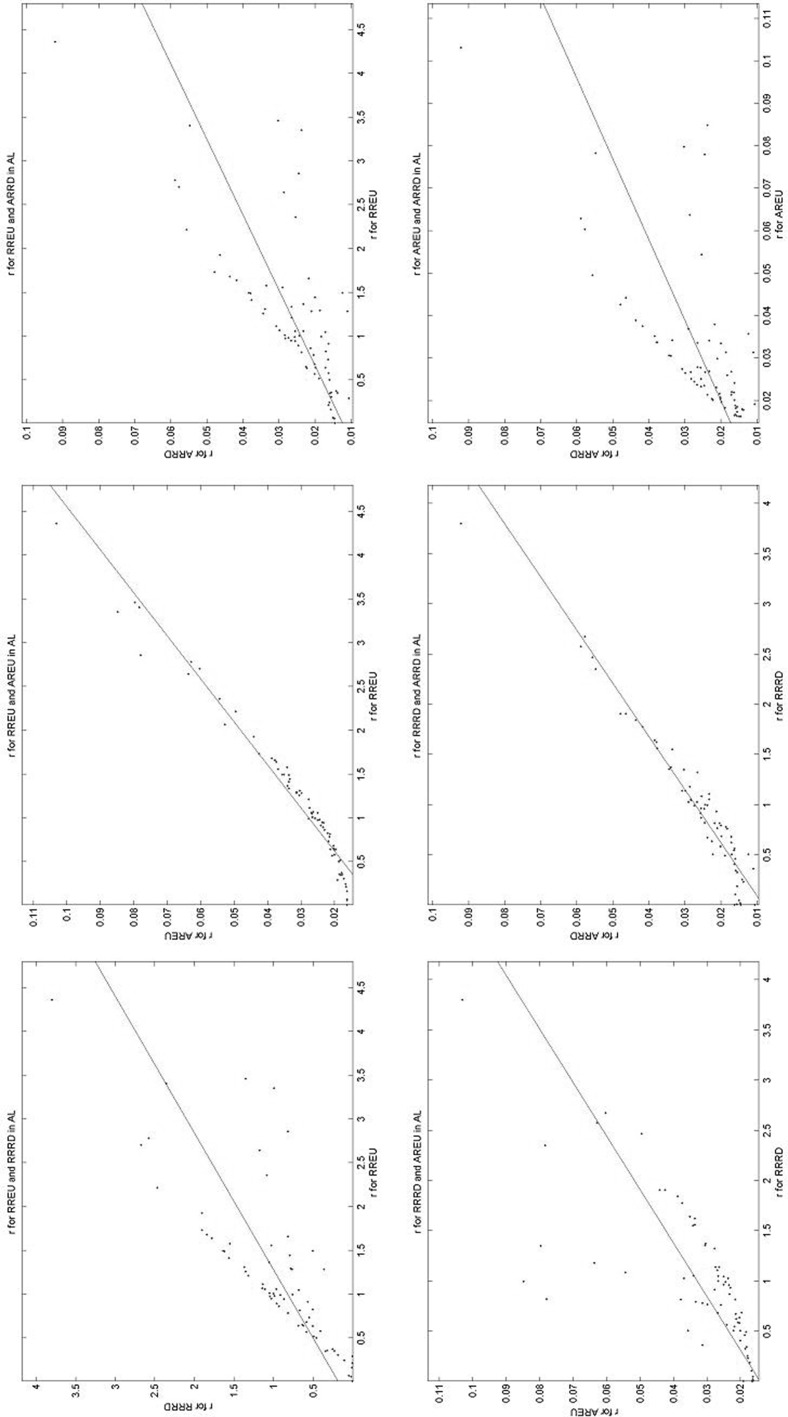
Estimates of *r* using AL across preference functionals. Each scatter plots the *r* value elicited using the AL method across the different preference functionals

We can also show the results graphically. We show here just a subset—the full set can be found in https://www.york.ac.uk/economics/exec/research/zhouandhey1/. Figure 6 shows the scatter of the estimated *r* values using the AL method across preference functionals. This figure suggests that getting the functional form wrong does not upset our estimation of the risk-aversion of the subjects. (Deck et al. (2008) also present such scatters and make the same point, though their risk-aversion indices are not estimated.) However, the figure of the estimated *g* values using the AL method across preference functionals (available in https://www.york.ac.uk/economics/exec/research/zhouandhey1/) suggests that if we get the utility function wrong then the estimate of the probability weighting parameter *g* may be quite seriously wrong.

The figure of the estimated *s* values using the AL method across preference functionals (available in https://www.york.ac.uk/economics/exec/research/zhouandhey1/) shows the scatters of the estimated *s* values using the AL method across the preference functionals. The scatters are almost always along the 45 degree line (as Table 4 shows). This means that, whether we assume RR or AR preferences, we get almost the same estimate of the noise in subjects’ responses.

We continue to analyse the different elicitation methods across preference functionals, and now consider those where the estimated parameters are not comparable. Table 5 gives the detail. Here we keep the elicitation method constant in each row and report the intercept, slope and correlation coefficient of the regression of an estimated parameter for each preference functional against the estimated parameter for each of the other preference functionals. Because of the lack of a relationship between the two parameters, other than monotonic increasing-ness, the obvious test to carry out is that the slope is positive; significance is good and this is reported in the *β* column. We also include a test of whether the intercept is significantly different from 0—as it should be. The results show that it is usually significantly different from zero.

### A comparison of the estimated parameters across elicitation methods

**Fig. 7:**
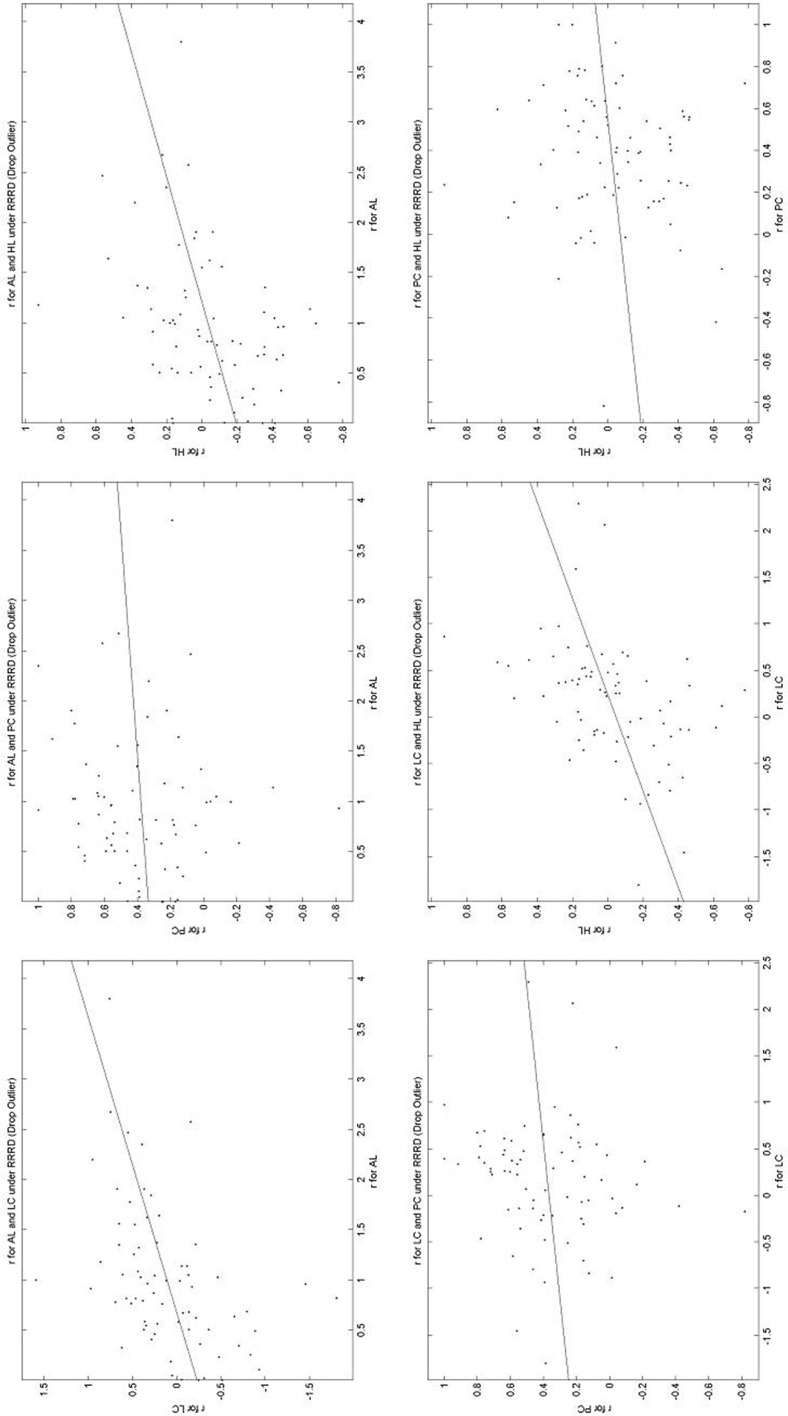
Estimates of *r* in RRRD across elicitation methods. Each scatter plots the *r* value elicited using the RRRD specification across the different elicitation methods

**Fig. 8:**
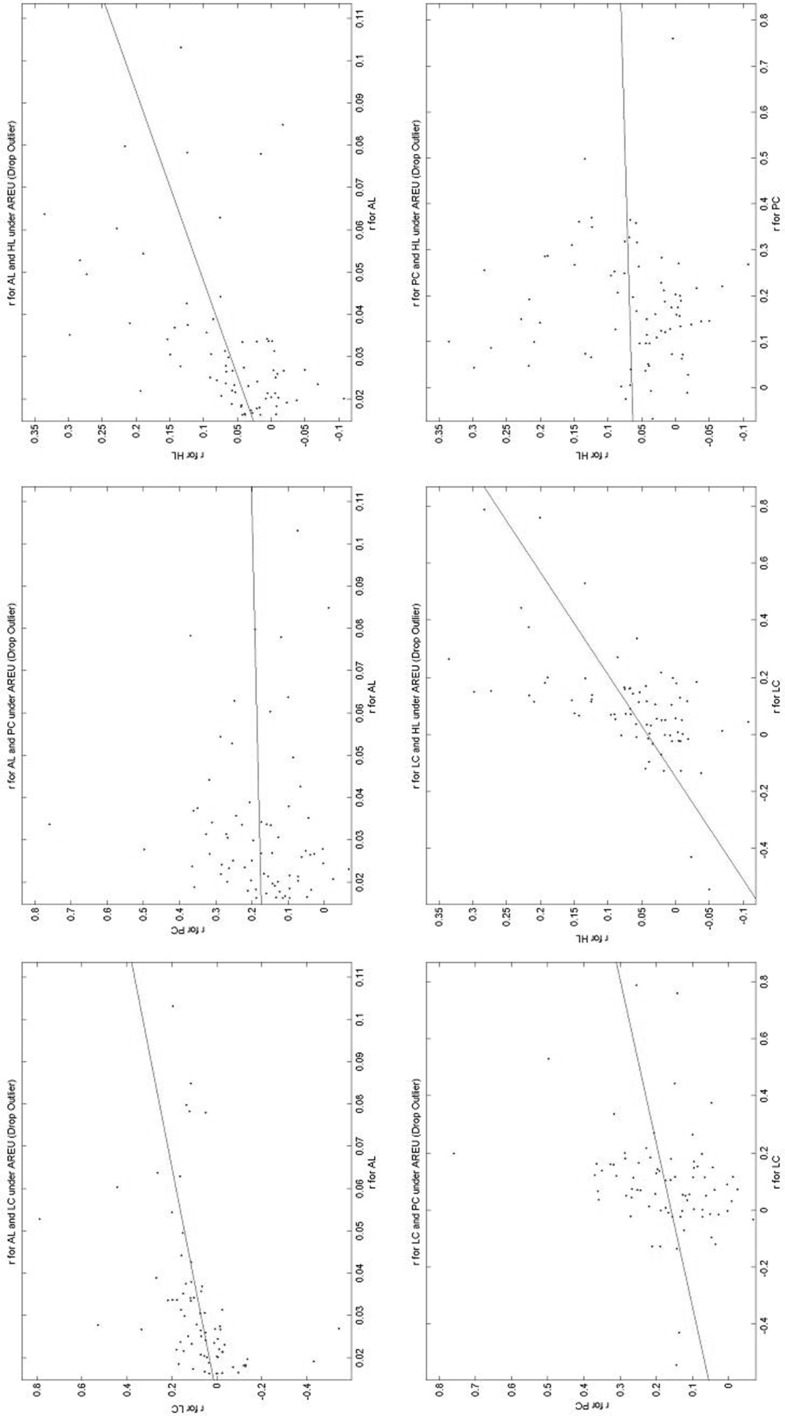
Estimates of *r* in AREU across elicitation methods. Each scatter plots the *r* value elicited using the AREU specification across the different elicitation methods

**Fig. 9:**
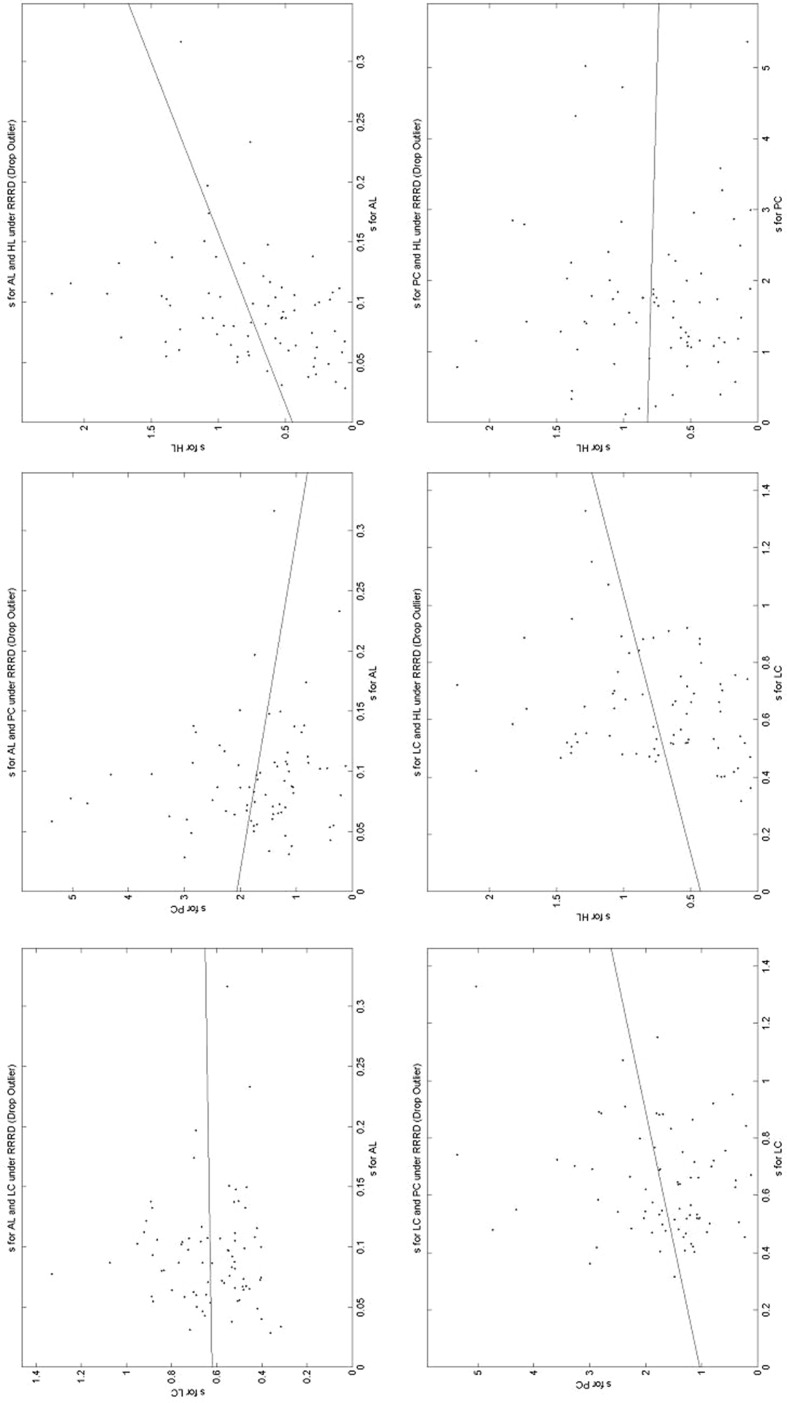
Estimates of *s* in RRRD across elicitation methods. Each scatter plots the *s* value elicited using the RRRD specification across the different elicitation methods

This is the central part of the paper. Here we compare the estimated parameters across elicitation methods. Table 6 gives a summary, while Figs. 7, 8 and 9 present a subset of graphical comparisons (the full set of 60 sets of comparisons can be found in https://www.york.ac.uk/economics/exec/research/zhouandhey1/). Let us start with Fig. 7 which shows the 6 scatters for the estimated *r* value for the RRRD functional; each scatter being the estimated values using one elicitation method plotted against the estimated values using another elicitation method, for all the non-excluded subjects. Figure 7 corresponds to rows 13–18 of Table 6. As the scales on the two axes differ, it helps to fit a regression line to the scatter. Table 6 gives the intercept (− 0.230) and slope (0.340) of this line. The intercept is significantly different from zero (at the 5% level) and the slope is significantly different from one (at 1%), as the asterisks indicate. As can be seen, the risk-aversion index elicited by AL is generally greater than that elicited by LC. This could result from what might be called a built-in bias with the allocation method—subjects tend to make allocations to avoid large differences in their payoff depending on which state occurs (thus encouraging risk-averse behaviour), while the BDM mechanism does not make so explicit the possible consequences of their actions. Indeed generally the risk-aversion elicited under allocation is generally higher than for the other three methods.

**Table 6:** A comparison of the estimated parameters across Elicitation Methods

Row	Parameter	PF	*x*	*y*	*α*	*β*	*ρ*
1	*r*	RREU	AL	LC	− 0.013	0.224***	0.417
2	*r*	RREU	AL	PC	0.484***	0.022***	0.073
3	*r*	RREU	AL	HL	0.003	0.132***	0.417
4	*r*	RREU	LC	PC	0.471***	0.098***	0.197
5	*r*	RREU	LC	HL	0.113***	0.253***	0.507
6	*r*	RREU	PC	HL	0.163**	0.038***	0.037
7	*s*	RREU	AL	LC	0.566***	0.240	0.056
8	*s*	RREU	AL	PC	1.589***	− 2.760*	− 0.157
9	*s*	RREU	AL	HL	0.554***	2.583	0.281
10	*s*	RREU	LC	PC	1.674***	− 0.549***	− 0.126
11	*s*	RREU	LC	HL	0.473***	0.517**	0.252
12	*s*	RREU	PC	HL	0.900***	− 0.074***	− 0.147
13	*r*	RRRD	AL	LC	− 0.230**	0.340***	0.426
14	*r*	RRRD	AL	PC	0.334***	0.046***	0.101
15	*r*	RRRD	AL	HL	− 0.194***	0.161***	0.377
16	*r*	RRRD	LC	PC	0.366***	0.060***	0.122
17	*r*	RRRD	LC	HL	− 0.045	0.193***	0.412
18	*r*	RRRD	PC	HL	− 0.071	0.129***	0.134
19	*s*	RRRD	AL	LC	0.620***	0.089*	0.022
20	*s*	RRRD	AL	PC	2.067***	− 3.640*	− 0.157
21	*s*	RRRD	AL	HL	0.445***	3.518**	0.322
22	*s*	RRRD	LC	PC	1.036**	1.080	0.196
23	*s*	RRRD	LC	HL	0.424**	0.557	0.212
24	*s*	RRRD	PC	HL	0.822***	− 0.014***	− 0.030
25	*g*	RRRD	AL	LC	0.916***	0.045***	0.048
26	*g*	RRRD	AL	PC	0.706***	0.365***	0.220
27	*g*	RRRD	AL	HL	0.602***	0.200***	0.138
28	*g*	RRRD	LC	PC	1.075***	0.030***	0.017
29	*g*	RRRD	LC	HL	0.764***	0.062***	0.040
30	*g*	RRRD	PC	HL	0.767***	0.052***	0.059
31	*r*	AREU	AL	LC	− 0.040	3.693***	0.391
32	*r*	AREU	AL	PC	0.171***	0.276	0.039
33	*r*	AREU	AL	HL	− 0.007	2.232**	0.468
34	*r*	AREU	LC	PC	0.159***	0.174***	0.252
35	*r*	AREU	LC	HL	0.042***	0.279***	0.592
36	*r*	AREU	PC	HL	0.064***	0.020***	0.029
37	*s*	AREU	AL	LC	0.493***	0.982	0.228
38	*s*	AREU	AL	PC	0.124***	0.032***	0.016
39	*s*	AREU	AL	HL	0.097***	− 0.124***	− 0.124
40	*s*	AREU	LC	PC	0.161***	− 0.061***	− 0.130
41	*s*	AREU	LC	HL	0.027*	0.107***	0.460
42	*s*	AREU	PC	HL	0.104***	− 0.122***	− 0.249
43	*r*	ARRD	AL	LC	− 0.098***	4.694***	0.415
44	*r*	ARRD	AL	PC	0.121***	0.656	0.060
45	*r*	ARRD	AL	HL	− 0.038*	2.369**	0.377
46	*r*	ARRD	LC	PC	0.129***	0.108***	0.128
47	*r*	ARRD	LC	HL	0.017*	0.200***	0.420
48	*r*	ARRD	PC	HL	0.026*	− 0.001***	− 0.002
49	*s*	ARRD	AL	LC	0.531***	0.911	0.217
50	*s*	ARRD	AL	PC	0.149***	0.020***	0.011
51	*s*	ARRD	AL	HL	0.114***	− 0.001***	− 0.001
52	*s*	ARRD	LC	PC	0.173***	− 0.037***	− 0.085
53	*s*	ARRD	LC	HL	0.034	0.134***	0.409
54	*s*	ARRD	PC	HL	0.137***	− 0.153***	− 0.201
55	*g*	ARRD	AL	LC	0.928***	0.104***	0.078
56	*g*	ARRD	AL	PC	1.078***	0.042***	0.024
57	*g*	ARRD	AL	HL	0.665***	0.065***	0.084
58	*g*	ARRD	LC	PC	1.132***	− 0.019***	− 0.015
59	*g*	ARRD	LC	HL	0.612***	0.115***	0.196
60	*g*	ARRD	PC	HL	0.720***	0.010***	0.021

The hypotheses being tested are *α* = 0 and *β* = 1

Here the parameters are comparable. The *α* (intercept) and *β* (slope) values are obtained from a regression of the estimated parameter value for the *y* preference functional against the estimated parameter value for the *x* preference functional. The *ρ* value is the correlation coefficient. If they produce the same estimates *α* should be zero and *β* should be unity

Key: see key for Table 4

* Significantly different at 10%; ** at 5% and *** at 1%

Relative to the others, the comparison discussed above (*r* for RRRD across elicitation methods) is one of the better comparisons. Examine Fig. 8 which shows the 6 scatters for the estimated *r* value for the AREU functional (corresponding to rows 31–36 of Table 6). Here, as Table 6 shows, 4 of the 6 intercept values are significantly different from zero and 4 of the 6 slope values significantly different from 1, so the different elicitation methods are generally leading to significantly different estimates. Once again the allocation method seems to be inducing more risk-averse behaviour. Figure 9, comparing different estimates of the precision parameter *s* for RRRD across the different methods (corresponding to rows 19–24 of Table 6) shows that sometimes the relationship is negative. Here the precision seems to be lower with the allocation method, and possibly highest on the Pairwise Choice method, though a direct comparison does not make much sense as the error on PC is on the utility difference (between the two lotteries) while the error on AL is on the difference in the amounts of implied money.

The figure of the estimated *g* values in RRRD across elicitation methods (corresponding to rows 25–30 of Table 6, available in https://www.york.ac.uk/economics/exec/research/zhouandhey1/) is arguably the worst, usually showing very little relationship between the elicited *g* values for RRRD. Here the *g* value for the LC method seems to have the largest variation in the estimated values and that for PC having the smallest.

Finally, even though the different elicitation methods seem to disagree on the estimates of the parameters, we should ask whether at least they agree on the best-fitting preference functional. Table 7 gives the results, with the criterion for the ‘best-fitting’ functional being either the raw log-likelihood, or either the Akaike or Bayes information criterion (both of which correct the log-likelihood for the number of parameters involved in the fitting). Table 7 shows that correcting for degrees of freedom does make a big difference. But here again, the different elicitation methods disagree: it is clear that AL puts the AR specifications first, while the other methods suggest that RR fits better. Moreover there is no general agreement as to which of EU and RD is the best.

**Table 7:** Best-fitting preference functional

Method	PF	LL	BIC	AIC
PC	RREU	1	25	33
	RRRD	38	20	12
	AREU	1	10	14
	ARRD	37	21	17
AL	RREU	1	0	1
	RRRD	74	2	2
	AREU	0	24	37
	ARRD	2	50	36
LC	RREU	2	14	18
	RRRD	49	14	12
	AREU	0	17	22
	ARRD	27	31	24
HL	RREU	1	40	40
	RRRD	49	6	6
	AREU	0	19	22
	ARRD	27	11	8

Key: columns: PF: the preference functional, LL: based on the raw log-likelihood, BIC: based on the Bayesian Information Criterion which is *k ln*(*n*) − 2*LL*, AIC: based on the Akaike Information Criterion which is 2 *k* − *2LL*

## Qualifications

We have explored one dimension of the key issue of how ‘best’ to elicit the risk attitudes of subjects—though the issue is clouded by the fact that we do not, and cannot, know the ‘true’ risk attitude of our subjects. This is, of course, assuming that there *is* a risk-attitude out there to measure. There are some who think that risk attitude varies with the context, and it may be that which we are picking up. If that is true, then, if one is planning to use the elicited risk attitudes to explain behaviour in some economic problem, it would seem sensible to elicit the risk attitude in the same context as the economic problem. The simplest way of doing this is by eliciting the risk attitude from behaviour in the economics problem itself—that is, by finding the risk attitude which best explains their behaviour in that problem. So the risk attitude is elicited as a by-product of studying behaviour in some economic problem.

If, however, one believes that there *is* a risk attitude out there, one can criticise what we have done on other grounds. There are several dimensions to the problem. We have studied two: the elicitation method and the functional specification. But there is also the dimension of the stochastic assumptions used when analysing the data. As Wilcox (2008) points out “choices of stochastic models may be far more consequential than choices of structures such as expected utility or rank-dependent utility.” But notice that he is comparing different functionals (which is what we do) and not different elicitation mechanisms. In that paper he shows that, when using the pairwise choice method for eliciting risk attitudes, the stochastic assumptions can lead to markedly changed elicited values. Once again one does not know the ‘true’ risk attitude, so one cannot declare one stochastic specification to be the ‘best’. Chapter 6 of Bardsley et al. (2010), Blavatskyy and Pogrebna (2010) and Stott (2006) make the same point. Wilcox (2008) looks solely at pairwise choice problems. He considers a variety of stochastic specifications, most varying in their heteroscedasticity. Our stochastic specifications are all homoscedastic. Given the different nature of our methods, it is clearly not the case that we can apply the same set of heteroscedastic models to each method, so it is not clear what comparisons one can make. But to go some way to meeting this criticism we have refitted our allocation data with an heteroscedastic specification—a Beta distribution.^21^ The figure of the results can be found in https://www.york.ac.uk/economics/exec/research/zhouandhey1/. In the figure, the left-hand graph is a scatter of the risk-aversion estimates for RREU obtained using the allocation method with a beta stochastic specification against that with the normal specification that we have used. The right-hand graph is the corresponding graph for RRRD. It will be seen that the estimates do differ a little with the stochastic specifications but the correlations are high (0.985 and 0.933 respectively).

So there are variations in the estimates produced with the different elicitation methods, and (smaller?) variations with different stochastic specifications. The latter may cancel out the former, and indeed it may be the case that with each pair of elicitation methods there is a pair of stochastic specifications for which the same estimates are produced. This is unlikely but suggests an interesting project for future research.

## Conclusions

One clear conclusion that emerges from our results is that the elicitation method—the context—*does* matter to the estimated risk-aversion index: there are big differences in the estimated risk attitudes across the elicitation methods. The choice of the preference functional seems to be less important. The choice of the utility function seems to be even less important.

This appears to send a clear message: risk-aversion should be elicited in the context in which it is to be interpreted. This suggests that one should estimate the risk-aversion index along with the other parameters of the model being fitted to the data; eliciting them in another context could lead to mis-interpretations of the data. As Loomes and Pogrebna (2014) write “In the short run, one recommendation is that researchers who wish to take some account of and/or make some adjustment for risk attitude in their studies should take care to pick an elicitation procedure as similar as possible to the type of decision they are studying…”. We would even go as far as suggesting modifying “as similar as possible” to “in the same decision problem”. To give an example, in an experiment on First price auction behaviour, testing whether subjects are following the Nash-optimal risk-averse strategy, one can find the risk-aversion which best explains the bidding decisions of the subjects. This is effectively what Isaac and James (2000) do.

In summary, our results suggest something that has been found elsewhere (largely in the psychological literature): namely, that subjects do not have a stable preference functional for making decisions under risk. One thing that could be done, as we have hinted above, is to investigate more carefully the stochastic component of decision-making, and make the stochastic specifications specific to the method. Or we could take up Loomes and Pogrebna’s call to understand better “how contextual or procedural factors interact with that process [of decision-making].” This is an important point. It suggests that economists should look at the *process* of decision-making, try and understand *how* people take decisions, and come up with behaviourally plausible preference stories relevant to particular elicitation methods. This suggests that risk-aversion might only have a meaning with a particular elicitation method, or, as we put it, in a particular context.

Payne et al. (1992) make a similar point when they argue that “preferences are often constructed rather than merely revealed”: that the strategy used to make a decision can be affected by the characteristics of the decision problem. They suggest that the selection of the strategy may be based on the tradeoff between effort and accuracy. This might be one possible explanation of our findings: for complicated elicitation methods, subjects might not be willing to expend sufficient effort to work out the ‘correct’ solution. In contrast, for simple elicitation methods, where less effort is required, subjects are more likely to reveal their ‘true preferences’. This point is echoed by Sher and McKenzie (2006), who argue that the ‘frame’ (the elicitation method) may affect decision-making, and by Hsee (1996), who reinforces Payne et al.’s point above. In our terminology, the context, or the frame, or the elicitation method, may affect decisions, and hence matter.

## Footnotes

1 There is a problem with those subjects who switch more than once—as we shall discuss later.

2 This triangle is used to represent lotteries over a set of three outcomes. Two of the probabilities in the lotteries are plotted on the vertical and horizontal axes, while the third is the residual from one. The points at the vertices are certainties while points properly inside the triangle are lotteries.

3 Though this is not necessarily true with other preference functionals.

4 The quotation marks are there because in an experimental setting it is impossible to provide completely continuous choice.

5 This is used when trying to elicit preferences over ambiguity.

6 Since we analyse the data across all subjects, possible order effects are cancelled out.

7 We note that this is a different presentation than that used by Loomes and Pogrebna (2014). Theirs is more appropriate in their setting; ours in ours as we wanted finer divisions (in steps of 0.01 rather than 0.1).

8 In contrast Loomes and Pogrebna had just 5 such lists. We used more because pre-experimental simulations indicated that to get accuracy in estimation (which is a different objective to that in Loomes and Pogrebna) we needed more.

9 In contrast Loomes and Pogrebna (2014) allowed subjects to switch at several points—but they write “the proportion of inconsistent participants in our data set ranged between 1.1 and 5.6%”. It is not immediately obvious how to analyse the data from such subjects.

10 Again these were chosen after pre-experimental simulations; the objective being to get a set of problems which would enable us to identify accurately the preference functional and its parameters.

11 Again these were chosen after pre-experimental simulations.

12 This works, as before, with Expected Utility preferences and may work with other preferences.

13 In interpreting this figure, note that on the horizontal axis is the number of the disk drawn randomly from a bag containing disks numbered from 1 to 100, and the vertical axis displays the corresponding payoff. As the numbers on the horizontal axis are uniformly distributed so are those on the vertical axis. It should not be interpreted as a probability density function.

14 Loomes and Pogrebna (2014) had just 13, but theirs were over three states and the exchange rates were always 1–1. We adopted a two-way allocation with non-unitary exchange rates partly because it is easier for subjects to understand, but more crucially because the econometric analysis of the data is simpler. The number of problems was again chosen after pre-experimental simulations.

15 By this ‘we’ mean when using our methodology; that of Loomes and Pogrebna and others differs. We have already discussed some of these in Sect. 3.

16 We could additionally fit a *mixture model* to the data, thus estimating *distributions* of the relevant parameters over all subjects, but this would miss the point as we want to investigate subject-by-subject estimated risk attitudes across elicitation methods.

17 This implies particular levels of risk-aversion.

18 The problematic parameter was often the *g* in the probability weighting function for RD. We imposed a lower limit of 0.3 (below which the weighting function is not monotonically increasing, and an upper limit which varied from subject to subject.

19 Note that the *r* value for the RR specifications cannot be compared with the *r* value for the AR specifications.

20 This sub-section can safely be skipped for those solely interested in the comparison across elicitation methods.

21 We assume that the actual allocations, instead of being normally distributed and centred on the optimal allocations, have a beta distribution centred on the optimal allocations. Specifically we have used *x**(1 − *s*) and *(1* − *x*)s* as the parameters of the Beta distribution (where *x** denotes the optimal allocation) which ensures that the mean is *x** and the variance *x*(1* − *x*)*/*s*.
